# Detection of *Helicobacter hepaticus* in Human Bile Samples of Patients with Biliary Disease

**DOI:** 10.1111/j.1523-5378.2009.00729.x

**Published:** 2009-12

**Authors:** Toshihide Hamada, Kenji Yokota, Kiyoshi Ayada, Kazuyuki Hirai, Tomoari Kamada, Ken Haruma, Kazuaki Chayama, Keiji Oguma

**Affiliations:** * Department of Gastroenterology, Miyoshi Central HospitalMiyoshi, Hiroshima; † Graduate School of Health ScienceOkayama; ‡ Department of Bacteriology Graduate School of Medicine, Dentistry, Pharmaceutical Science, Okayama UniversityOkayama; § Division of Gastroenterology, Department of Internal Medicine, Kawasaki Medical SchoolKurashiki; ¶ Department of Medicine and Molecular Science, Graduate School of Biomedical Sciences, Hiroshima UniversityHiroshima, Japan

**Keywords:** *Helicobacter hepaticus*, bile, gallbladder stone, cholecystitis

## Abstract

**Background:**

Since the discovery of *Helicobacter pylori*, various enterohepatic *Helicobacter* spices have been detected in the guts of humans and animals. Some enterohepatic Helicobacters have been associated with inflammatory bowel disease or liver disease in mice. However the association of these bacteria with human diseases remains unknown.

**Materials and Methods:**

We collected 126 bile samples from patients with cholelithiasis, cholecystitis, gallbladder polyp, and other nonbiliary diseases. Samples were screened for the presence of enterohepatic *Helicobacter* spp. using cultures, nested PCR, or in situ hybridization. We tested for antibodies to *H. pylori* and *H. hepaticus* by Western blot analysis.

**Results:**

Attempts at cultivation were unsuccessful. However, *H. hepaticus* was detected in bile samples with nested PCR whereas *H. bilis* was not. *Helicobacter hepaticus* in the bile was confirmed by in situ hybridization, but *H. hepaticus* from bile samples was coccoid in appearance. We detected immunoglobulin G antibodies to *H. hepaticus* in bile samples by Western blotting. *Helicobacter hepaticus* was detected in 40 (32%) of total 126 samples as *H. hepaticus* positive if at least one of the three methods with nested PCR, in situ, or Western blotting. Patients with cholelithiasis (41%) and cholecystitis with gastric cancer (36%) had significantly higher (*p* = .029) prevalence of *H. hepaticus* infection than samples from patients with other diseases.

**Conclusion:**

*Helicobacter hepaticus* may closely associate with diseases of the liver and biliary tract in humans.

Enterohepatic Helicobacters have been shown to inhabit the intestine and hepatobiliary tracts and may be associated with a variety of diseases [1]. The enterohepatic Helicobacters were first recognized in laboratory rodents and have been considered a component of the resident microbiota, or normal flora. However, the role of enterohepatic Helicobacters in human diseases remains obscure.

*Helicobacter hepaticus*, perhaps the most studied member of enterohepatic *Helicobacter*, colonizes the lower gastrointestinal tract, including the cecum, colon, and hepatobiliary system of mice [2–4]. *Helicobacter hepaticus* infection can cause chronic active hepatitis and typhlocolitis in immunocompetent mice and can lead to liver carcinoma in male mice of susceptible strains [5–9]. Natural and experimental infection with *H. hepaticus* in certain immunodeficient mice can induce inflammatory bowel disease [10,11]. *Helicobacter bilis* was also identified in inbred mice with chronic hepatitis [12]. *Helicobacter bilis* infection in immunodeficient rodents causes typhlocolitis and diarrhea [13]. These studies have prompted the increased use of murine models with *H. hepaticus* infection to elucidate the possible roles of *Helicobacter* in the development of gastrointestinal diseases in humans. *Helicobacter hepaticus* or *H. bilis* infection in humans was reported. For example, patients with chronic liver disease have been reported to have significantly higher levels of antibody to *H. bilis* and *H. hepaticus* compared to healthy subjects [14]. Although the *H. pylori* urease A gene (nested amplicon) has been frequently detected in bile samples, to date *H. bilis-*16S rRNA genes have only been detected in two cases, and *H. hepaticus* has not been detected [15]. These papers indicate that enterohepatic *Helicobacter* spp. may also infect in the human.

The aim of this study was to directly detect enterohepatic *Helicobacter* spp. from human bile samples. We cultured bile samples taken from patients with and without hepatobiliary diseases to determine whether *Helicobacter* spp. were present. We used PCR and nested-PCR for detecting *H. hepaticus* or *H. bilis*. As nested-PCR for only *H. hepaticus* was positive, we tested the bile samples for in situ hybridization by a probe for *H. hepaticus* and for Western blotting by anti-*H. hepaticus* antibodies.

## Materials and Methods

### Bile Samples

This study was approved by the Regional Ethics Committee of Miyoshi Central Hospital and all patients provided informed consent. The final clinical diagnoses of the patients were based on clinical imaging findings including endoscopic retrograde cholangiopancreatography, ultrasonogram, and computed tomography or surgery. Diagnoses included cholelithiasis (n = 60; mean age 61.2 years from 26 men and 34 women), cholecystitis and gastric cancer (n = 28; mean age 71.8 years from 19 men and 9 women), gallbladder polyp (n = 6; mean age 57.2 years from one man and five women), or other nonbiliary diseases (n = 32; mean age 69.2 years from 17 men and 15 women). We collected a total 126 bile samples from 63 men and 63 women (mean age 65.5 years) who underwent surgery, endoscopic retrograde cholangiopancreatography, percutaneous transhepatic cholangiography and drainage, or percutaneous transhepatic gallbladder drainage. Bile samples were stored at −80 °C until processing and testing.

### Bacterial Strains

*Helicobacter pylori* (ATCC 43504, American Type Culture Collection) were cultured on brain heart infusion (BHI) agar supplemented with 7% sterile defibrinated horse blood at 37 °C in a microaerobic chamber (10% CO_2_, 5% O_2_, and 85% N_2_). *Helicobacter hepaticus* (ATCC 51448) and *H. bilis* (ATCC 51630) were cultured under microaerobic conditions using GasPak Plus Hydrogen + CO_2_ system without catalyst (3% H_2_, 10% CO_2_, 5% O_2_, 82% N_2_) (BD JAPAN, Tokyo, Japan). The morphology (coccoid or rod form) of *H. pylori* was observed following culture for a period of 3 days to 2 weeks using Gram staining. Due to their slow growth rates, the morphology of *H. hepaticus* and *H. bilis* (coccoid or rod form) were observed using Gram staining following culture for between 1 and 4 weeks.

### Cultures

Bile samples (1 mL) were passed through a Millipore Filter (0.45 μm pore size, 47 mm diameter) (Millipore Filter Corp., Billerica, MA, USA) by suction. Based on the fact that ammonium sulfate has been shown to allow the coccoid form of *H. pylori* to go into the logarithmic phase [16] and that bovine serum albumin is a supplement used for the growth of *H. pylori* [17], the filter was washed under suction using 10 mL of 10 mmol/L ammonium sulfate, 10 mmol/L NaHCO_2_ containing 0.9% deoxycholic acid, or phosphate-buffered saline (PBS) containing 0.1% bovine serum albumin. Following washing the filters from all 126 bile samples were placed onto BHI blood agar plates and incubated at 37 °C in a microaerobic condition as previously described for 7 days. In addition, a portion of 5–10 μL of bile samples was directly inoculated and incubated on a BHI blood agar plate.

### PCR Amplification

DNA was purified from 100 μL of bile sample using MagExtractor (TOYOBO Co. Ltd, Osaka, Japan). Primers for *H. bilis, H. hepaticus, and Helicobacter* universal primers were designed by modification of primers which were previously reported from Goto et al. [18] (Table 1). PCR reactions were conducted in an iCycler^R^ (BioRad, Hercles, CA, USA). Reaction mixtures (50 μL of total volume) contained 1 μmol/L of oligonucleotide primer, 1× PCR buffer (*EX Taq* buffer), 1 U of *EX Taq* polymerase (TAKRA Bio Inc., Osaka, Japan), and 100 ng of template DNA, unless otherwise stated.

**Table 1:** Oligonucleotide primers used to amplify 16S rRNA gene

Primer		Sequence^a^	Product size
*Helicobacter* universal^b^	Forward	CTATGACGGGTATOCGGC	781 bp
	Reverse	CTCACGACACGAGCTGAC	
*H. hepaticus*	Forward	GAAACTGTTACTCTG	405 bp
	Reverse	TCAAGCTCCCCGAAGGG	
*H. bilis*	Forward	CAGAACTGCATTTGAAACTAC	418 bp
	Reverse	AAGCTCTGGCAAGCCAGC	

a Nucleotide sequences for *H. hepaticus* and *H. bilis* were cited from GenBank accession number L39122 and U18766, respectively.

b Positions of *Helicobacter* universal primers were correspond to *H. hepaticus* 16S rRNA sequence positions at 254–271 for forward primer and at 1018–1035 for reverse primer.

Nested PCR was performed on template DNA from bile samples using *H. bilis* or *H. hepaticus* specific primers. The second PCR primers were designed to lie within the PCR products of the *Helicobacter* universal primers. The first PCR reaction using *Helicobacter* universal primers for the DNA samples were preheated at 98 °C for 2 minutes and then subjected to 30 cycles consisting of denaturation at 98 °C for 10 seconds, primer annealing at 55 °C using *Helicobacter* universal primers for 30 seconds, and extension at 72 °C for 1 minute. The second PCR reaction individually used primers for *H. hepaticus* or *H. bilis*, with 5 μL of PCR products from the first PCR, was preheated at 98 °C for 2 minutes and then subjected to 30 cycles consisting of denaturation at 98 °C for 10 seconds), primer annealing at 55 °C using *H. hepaticus* primers or *H. bilis* primers for 30 seconds, and extension at 72 °C for 30 seconds. Samples of 10 μL were separated by electrophoresis on a 2% of NuSieve agarose gel (FMC Bioproducts Inc., Rockland, ME, USA), stained with ethidium bromide, and visualized under ultraviolet light.

### In situ Hybridization

In situ hybridization was performed on nylon membrane using a 3′-Cy3 labeled DNA probe (GAA ACT GTT ACT CTG GAG TGT GGG AGA GGT) for position 575–604 of the 16S rRNA gene of *H. hepaticus*. Bile samples (100 μL) were diluted to 10-fold with PBS and were adhered by suction to the nylon membrane using the Bio-Dot™ vacuum blotter (BIO-RAD). The membrane was prehybridized with calf thymus DNA in hybridization buffer containing 5× SSPE (0.2 mol/L phosphate buffer, pH 7.4, 2.98 mol/L sodium chloride, 0.02 mol/L EDTA), 5× Denhalrdt’s solution, and 0.5% SDS for 1 hour. Hybridization with the probe was performed at 60 °C for 18 hours in a water bath. After hybridization, the membrane was washed three times in 0.2× SSPE containing 0.1% SDS (each wash for 15 minutes at 65 °C) to remove a nonspecific binding. The membrane was observed and photographed using a Leitz Vario-Orthomat Camera System (Ernst Leitz, Wetzlar, Germany).

### Western Blotting

To prepare for cell lysates for Western blot analysis, bacteria were cultured in BHI medium with 5% horse serum in a microaerobic jar with continuous shaking for 5 days for *H. pylori* and 10 days for *H. hepaticus*. Cells were collected by centrifugation at 6400 × *g* for 20 minutes. Bacterial cells were washed with PBS and lysed by ultrasonication by ASTRASON W-380 ultrasonic processor (Heat System-Ultrasonics Inc., Farmingdale, NY, USA) in distilled water containing 1 mmol/L of phenylmethylsulfonyl fluoride for 20 minutes. The cell sediment was centrifuged at 44,280 × g for 30 minutes, and then the supernatant was used for Western blot analysis.

Western blotting was performed as previously described [19]. Bacterial lysates (1 mg/mL) of *H. pylori* or *H. hepaticus* were subjected to SDS-PAGE and transferred to PVDF membranes (Millipore Filter Corp.). The membranes were incubated with the 126 bile samples (1 : 100 dilutions) followed by peroxidase-labeled rabbit anti-human immunoglobulin G (IgG) antibody (DAKO, Japan, Tokyo) as secondary antibody. Blots were visualized using ECL™ Western blotting detection reagents (GE Healthcare Japan, Tokyo). The luminol reaction was detected with a LAS-1000mini Bio-Imaging Analyzer System (Fuji Photo Film Co., Tokyo).

### Survival of Viable *H. hepaticus* in Bile Samples

*Helicobacter hepaticus* (approximately 1 × 10^6^ CFU/mL in 50 μL) were inoculated into a 24-well culture plate with 450 μL of PBS, 0.9% deoxycholic acid in PBS, or in human bile samples obtained from three different persons. Bacteria were incubated at 37 °C in 10% CO_2_ incubator under100% humidity. Following incubation for 1, 3, 6, and 24 hours, 10μL samples from each well were inoculated onto BHI agar containing 5% horse blood. Plates were incubated at 37 °C in microaerobic condition using GasPak Plus Hydrogen plus CO_2_ system without catalyst for 7 days, and the colonies were counted. Two independent experiments were conducted with each sample being tested in triplicate.

### Statistical Analysis

The association between *H. hepaticus* infected samples by patient disease type (cholelithiasis, cholecystitis with gastric cancer, other diseases) was tested with the chi-squared test statistic. A *p*-value <0.05 was considered significant.

## Results

### Culture

After 7 days incubation, the surface of the cultured membrane was scraped and Gram staining was performed. However no spiral shaped Gram-negative bacteria were observed by microscopy. In addition, cells from the membrane were subcultured onto BHI blood agar plates and incubated under microaerobic conditions at 37 °C for 7 days. The majority of colonies that grew on these plates were *Escherichia coli* or *Enterococcus* spp. Suspicious colonies which looked like members of the *Helicobacter* genus were further subcultured onto BHI blood agar; however, no enterohepatic *Helicobacter* were cultured.

### PCR

**Figure 1:**
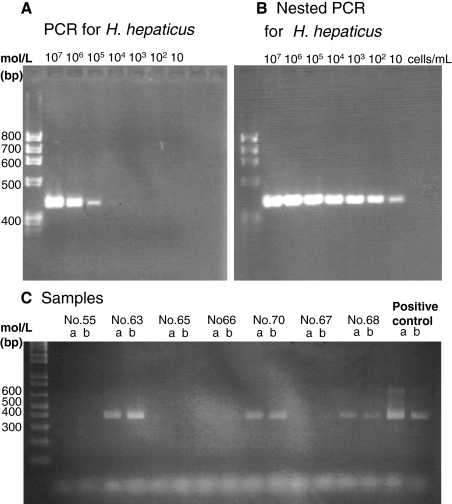
Detection of *Helicobacter hepaticus* DNA in bile samples *A* and *B*: sensitivity and specificity of nested PCR were confirmed by DNA which was purified from serial dilution of cultured *H. hepaticus*. (*A*) *Helicobacter hepaticus* DNA was detected in samples with >10^5^ cultured cells/mL by PCR using *hepaticus*-specific primers as a positive control. (*B*) Nested-PCR was performed on same samples. *H. hepaticus* DNA was detected in samples contained with <10 cultured cells/mL by nested PCR when we employed for the first PCR using *Helicobacter* universal primers and for the second PCR using *hepaticus* specific primers. (*C*) *Helicobacter hepaticus* DNA purified from bile samples was detected by nested PCR. PCR reactions were curried out two concentrations of DNAs, sample b is diluted sample a by 10 times, purified from bile samples. Positive cases for *H. hepaticus* were no. 63, no. 68, no. 70. Positive controls were shown in right side.

We prepared three sets of primers: *Helicobacter* universal*, H. hepaticus, and H. bilis* (Table 1). To confirm the specificity of the primers, DNA purified from *H. pylori, H. hepaticus,* and *H. bilis* was amplified using these three primer sets. When universal *Helicobacter* primers were used a 781-bp fragment was amplified from template DNA obtained from *H. pylori*, *H. bilis,* and *H. hepaticus*. The primer sets for *H. bilis* and *H. hepaticus* were both shown to be specific amplifying a 418-bp and a 458-bp fragment, respectively (data not shown). The sensitivity of the *H. hepaticus*-specific primers was confirmed. DNA was extracted from serial dilution of a 100-μL sample of *H. hepaticus* culture (10^7^ to 10 cells/mL in TE buffer) by boiling at 100 °C for 5 minutes. The DNA was amplified by PCR using *H. hepaticus* primers. PCR products were detected in samples that contained >10^5^ cells/mL (Fig. 1A). In addition, we performed nested PCR first using *Helicobacter* universal primers followed by *H. hepaticus*-specific primers. Using nested PCR, we were able to detect *H. hepaticus* purified DNA from samples containing <10 cells/mL (Fig. 1B). The sensitivity of the *H. bilis* PCR was the same as that reported for the *H. hepaticus* PCR (data not shown). To detect *H. hepaticus* and *H. bilis* in bile samples PCR was conducted using *Helicobacter* universal primers as well as the *H. hepaticus* or *H. bilis* primers. Eight samples were positive for the first PCR using *Helicobacter* universal primer, but no products were observed by first PCR using *H. hepaticus* or *H. bilis-*specific primer. Nested PCR on the PCR products of the *Helicobacter* universal primers using the *H. hepaticus* or *H. bilis* primers detected only *H. hepaticus* DNA, with 16 of the 126 bile samples being positive for *H. hepaticus* (Table 2). Examples of positive and negative samples in the PCR are shown in Fig. 1C.

**Table 2:** Number of *Helicobacter hepaticus* positive samples in 126 samples by three different methods

Method (s)	Number of positive samples (%)
PCR	16 (12.7)
In situ	25 (19.8)
WB^a^	17 (13,5)
Double positive with PCR and In situ	10 (7.9)
Double positive with PCR and WB	4 (3.1)
Double positive with in situ and WB	7 (5.6)
Triple positive with PCR and in situ and WB	2 (1.6)
Positive with one method by PCR or in situ or WB	40 (32)

a Western blotting.

These positive samples did not correspond to the eight samples which were positive by PCR using *Helicobacter* universal primers. PCR products of *H. hepaticus* (405 bp) were sequenced and confirmed to be 100% identical to *H. hepaticus* 16S ribosormal RNA sequence.

### In situ Hybridization

**Figure 2:**
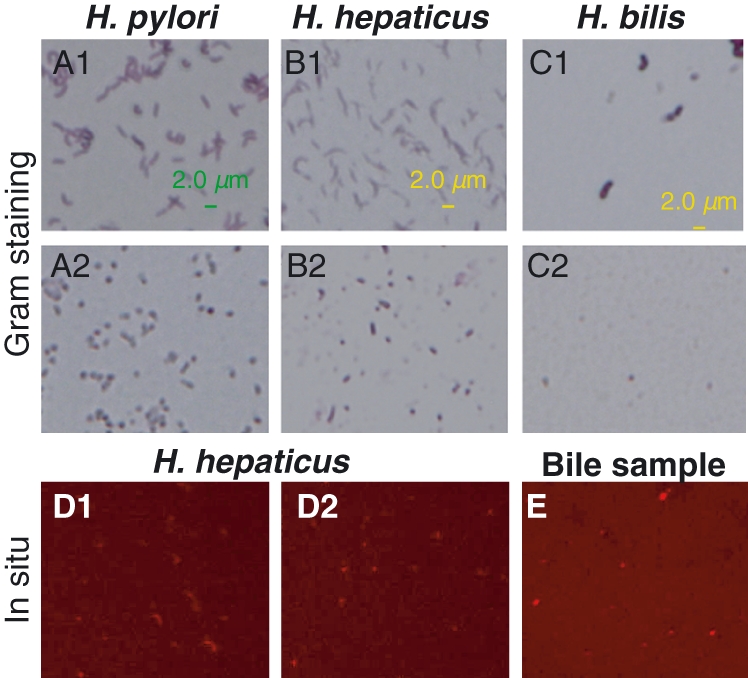
*Helicobacter hepaticus* was detected by in situ hybridization. Morphology was observed after Gram staining of *H. pylori* (*A*1: 5 day culture and *A*2: 2 week culture), *H. hepaticus* (*B*1: 7 day culture and *B*2: 2 week culture) or, *H. bilis* (*C*1: 7 day culture and *C*2 2 week culture). Cultured *H. hepaticus* was adhered to a 0.45-mm nylon membrane (*D*1: 7 day culture and *D*2: 2 week culture as positive controls). Membranes were hybridized using a cy-3-labeled anti-*H. hepaticus* 16S ribosormal RNA probe was hybridized to bacterial RNA. Morphology was observed by a fluorescent microscopy. (*E*): A positive bile sample.

Morphology of *H. pylori* was observed by Gram staining after 5 days and 2 weeks incubation. As *H. hepaticus*, and *H. bilis* grow more slowly than *H. pylori*, morphologies of *H. hepaticus*, and *H. bilis* were observed after 7 days and 2 weeks. *Helicobacter pylori* retained its spiral morphology for 5 days (Fig. 2A1), however after 7 days incubation the coccoid form started to appear, and by 2 weeks all cells were in the coccoid form (Fig. 2A2). Morphologies of *H. hepaticus* and *H. bilis* kept spiral shaped within 10 days incubation (Fig. 2B1, C1). Coccoid forms of *H. hepaticus* and *H. bilis* were appeared after 2 weeks incubation. The coccoid forms of three bacteria had similar morphologies in three strains (Fig. 2A2, B2, and C2). The cultured bacteria were transferred to nylon membranes, and hybridized to an *H. hepaticus* probe. The *H. hepaticus* probe reacted with the logarithmic phase (spiral-shaped) bacteria and with coccoid forms of *H. hepaticus* (Fig. 2D1, D2). However the probe did not react with *H. bilis and H. pylori.* Bile samples were adhered to membranes and reacted with the probe. We observed positive reactions in 25/126 samples (Table 2). All of positive samples were in the coccoid form (Fig. 2E).

### Western Blotting

**Figure 3:**
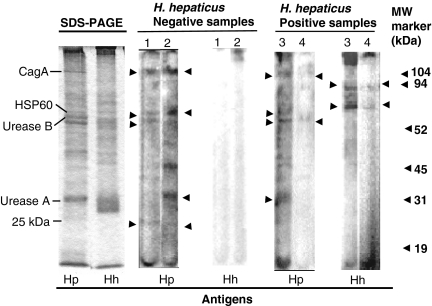
Anti-*H. hepaticus* and *H. pylori* antibodies in bile samples. Antibodies to *H. hepaticus* or *H. pylori* were detected by Western blot analysis. Bile samples were diluted 10-fold with PBS and reacting with *H. pylori* (Hp) and *H. hepaticus* (Hh) antigens. Protein profiles of Hp and Hh are shown by SDS-PAGE. Most of bile samples reacted to some of *H. pylori* antigens of CagA, HSP60, Urease A or B subunit, or 25 kDa protein when the samples were positive for *H. pylori* (arrows, samples no. 1, 3, 2, 4). However, *H. hepaticus* positive samples (arrows, samples no.3 and 4) reacting to 70 kDa and/or 90 kDa antigens of *H. hepaticus* (arrow).

Bile samples were reacted to *H. pylori* and *H. hepaticus* by Western blotting. Reactions were quite weak therefore it was difficult to assess positive reactions. Typical positive and negative cases for *H. hepaticus* or *H. pylori* were shown in Fig. 3. Positive samples for *H. pylori* reacted to several antigens (CagA, HSP60, urease A and B, or 25 kDa protein) of *H. pylori*. Major antigens were determined by their locations by specific antibodies to CagA (SANTA CRUZ biotechnology Inc., Santa Cruz, CA, USA), HSP60, Urease A, Urease B, 25 kDa antigen [19] (data not shown). Positive bile samples for *H. hepaticus* reacted to the 70 kDa and/or 90 kDa antigens of *H. hepaticus*. All samples in Fig. 3 were positive for *H. pylori* and they reacted some of major antigens of *H. pylori*. In no. 1 and no. 2 samples, no band was detected using *H. hepaticus* antigens. No. 3 and no. 4 were double positive for *H. hepaticus* and *H. pylori*. Seventeen bile samples in 126 samples were positive for *H. hepaticus* (Table 2) and 69 samples were positive for *H. pylori*.

### Survival of Viable *H. hepaticus* in Human Bile

**Figure 4:**
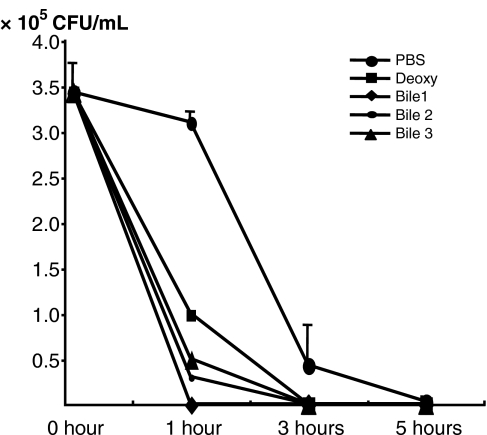
Survival viable *H. hepaticus* in the biles *H. hepaticus* (approximately 1x10^6^ CFU/mL in 50 μL) were incubated with 450 μL of PBS, 0.9% deoxycholic acid in PBS, or human bile samples, which were obtained from three different persons, at 1, 3 and 5 hours. CFU of *H. hepaticus* did not decrease for 1 hour in PBS. The viable bacteria markedly decreased in the PBS at 3 hours, and they were not detectable at 5 hours. Viable *H. hepaticus* in the bile samples were immediately decreased at 1 hour.

Survival of *H. hepaticus* in human bile was evaluated. After 1 hour, CFU of *H. hepaticus* in PBS had not decreased. However, CFU of *H. hepaticus* were markedly decreased after 3 hours, and no colony was detected after 5 hours. In contrast, CFU of hepaticus in both bile samples and 0.9% deoxycholic acid decreased after 1 hour (Fig. 4).

### Disease Specificity

The percentage of *H. hepaticus*-positive samples by each method (PCR, in situ, and Western blotting) are shown in Table 2. We determined samples as *H. hepaticus*-positive if at least one of the three methods is positive. The ratio of *H. hepaticus*-positive samples in various diseases was shown in Table 3. The percentage of patients that were *H. hepaticus* positive was significantly higher (*p* = .029) in those with cholelithiasis (41%) and cholecystitis and gastric cancer (36%) compared with those with gallbladder polyps (17%) and other diseases (13%).

**Table 3:** *Helicobacter hepaticus* positive samples in various diseases

Disease	Sample number	Positive sample^a^ number (%)
Cholelithiasis	60	25 (41)
Cholecystitis with gastric cancer	28	10 (36)
Gallbladder polyp	6	1 (17)
Other disease	32	4 (13)
Total	126	40 (32)

Totally 126 bile samples were collected in Miyoshi Central hospital.

a Positive ratio among various diseases was significantly (*p* = .029) different by chi-squared test.

## Discussion

In general culture of *Helicobacter* species and in particular nonpylori Helicobacters is difficult. Indeed, we are unaware of any reports of the culture of *Helicobacter* species from human bile. Despite prolonged incubation and washing the membrane adhered samples with buffer to prevent toxicity from deoxycholic acid and chenodeoxycholic acid all of our culture attempts were negative for *Helicobacter* species. Based on our in situ hybridization studies it was clear that the majority of *H. hepaticus* in bile samples were in the coccoid form and that the numbers present were low. Given this, we speculate that isolation of logarithmic phase of *H. hepaticus* from humans may require the use of substantial liver samples, gallbladder mucosa, or intestinal mucosa.

Seropositivity for *H. hepaticus* and *H. bilis* has been reported in humans. However, there is cross-reactivity among *H. bilis, H. hepaticus*, and *H. pylori*. Nilsson et al. showed elevated serum antibodies to *H. hepaticus* as measured by ELISA after a preabsorption step using *H. pylori* antigens, among patients with chronic liver disease [20]. Patients with chronic liver disease have increased antibody responses to *H. hepaticus* and *H. bilis* in patients compared to general population adults and blood donors, and cross-reactivity between *H. bilis* and *H. pylori* was also reported [21]. In this study, our detection of increased antibody responses to *H. hepaticus* with Western blotting analysis showed that IgG antibody to *H. hepaticus* was reacted with 90-kDa and/or 70-kDa bands in the positive samples. In contrast, positive cases for *H. pylori* reacted with some major *H. pylori* antigens which were detected as CagA, HSP60, Urease A and B subunit, or 25 kDa protein. Vorobjova et al. indicated that *H. hepaticus*-positive sera reacted with a 60-kDa band (heat shock protein) after absorption by *H. pylori* antigen [22]. Unfortunately, we could not measure serum antibodies, but this is similar to our results with bile samples and suggests that the IgG antibody in the bile may have originated from the serum.

The pathogenicity of *H. hepaticus* in humans remains obscure. Maurer et al. reported that mouse model of the cholelithiasis was induced by infection with enterohepatic *Helicobacter* spp. They reported that 40% of mice infected with *H. hepaticus* developed a cholesterol gallstone [23]. In our study, the prevalence of *H. hepaticus* infection in samples from patients with gallstones or cholecystitis was higher than in samples from patients with other diseases. Together these findings of both mice models and our human data suggest that *H. hepaticus* infection may contribute to the development of gallstones.

While *H. hepaticus* was detected in bile samples only a limited number were present and these were in the coccoid form. We showed that high concentration of deoxycholic acid in bile might be toxic to viable *H. hepaticus* (Fig. 4). Despite this, our results suggest that viable *H. hepaticus* may be able to infect the liver, gallbladder epithelium, or intestine of humans.

Although a high prevalence of *H. hepaticus* was detected in patients with cholelithiasis and cholecystitis we were unable to grow *H. hepaticus* from gallbladder bile samples. The mechanisms whereby gallstones develop are unknown but enterohepatic *Helicobacter* spp. may be closely associated with this disease in human.
